# Loss of Gadkin Affects Dendritic Cell Migration *In Vitro*

**DOI:** 10.1371/journal.pone.0143883

**Published:** 2015-12-01

**Authors:** Hannah Schachtner, Mirjana Weimershaus, Vanessa Stache, Natalia Plewa, Daniel F. Legler, Uta E. Höpken, Tanja Maritzen

**Affiliations:** 1 Molecular Physiology and Cell Biology Section, Leibniz-Institute for Molecular Pharmacology (FMP), Berlin, Germany; 2 Max-Delbrück-Center for Molecular Medicine (MDC), Berlin, Germany; 3 Biotechnology Institute Thurgau (BITg) at the University of Konstanz, Kreuzlingen, Switzerland; Istituto Superiore di Sanità, ITALY

## Abstract

Migration is crucial for the function of dendritic cells (DCs), which act as outposts of the immune system. Upon detection of pathogens, skin- and mucosa-resident DCs migrate to secondary lymphoid organs where they activate T cells. DC motility relies critically on the actin cytoskeleton, which is regulated by the actin-related protein 2/3 (ARP2/3) complex, a nucleator of branched actin networks. Consequently, loss of ARP2/3 stimulators and upstream Rho family GTPases dramatically impairs DC migration. However, nothing is known yet about the relevance of ARP2/3 inhibitors for DC migration. We previously demonstrated that the AP-1-associated adaptor protein Gadkin inhibits ARP2/3 by sequestering it on intracellular vesicles. Consistent with a role of Gadkin in DC physiology, we here report Gadkin expression in bone marrow-derived DCs and show that its protein level and posttranslational modification are regulated upon LPS-induced DC maturation. DCs derived from Gadkin-deficient mice were normal with regards to differentiation and maturation, but displayed increased actin polymerization. While the actin-dependent processes of macropinocytosis and cell spreading were not affected, loss of Gadkin significantly impaired DC migration *in vitro*, however, *in vivo* DC migration was unperturbed suggesting the presence of compensatory mechanisms.

## Introduction

Cell migration is essential for the functioning of the immune system. Dendritic cells (DCs) are a pivotal example for this fact due to their far apart lying places of action [1]. DC migration from the periphery to draining lymph nodes is crucial for the induction of an adaptive immune response against invading pathogens [2]. Immature DCs reside as sentinels for the detection of pathogens in exposed tissues such as skin and mucosal surfaces, where they continuously sample foreign antigens [1]. Pathogen encounter triggers DC maturation e.g. via Toll-like receptors, which includes an increase in the surface levels of the chemokine receptor CCR7 [3] as well as the upregulation of co-stimulatory molecules to efficiently prime T cells. Guided by gradients of the CCR7 ligands CCL21 and CCL19, DCs emigrate from the tissue interstitium and enter afferent lymphatic vessels to reach the draining lymph nodes [4]. Noteworthy, CCL21 seems to be more important for DC homing as mice lacking CCL19 show neither aberrant DC maturation nor migration deficits [5]. In lymph nodes, DCs present the processed antigen to naive T cells thereby selecting T cells carrying a cognate antigen receptor from the enormous T cell repertoire and inducing adaptive immunity. Hence, DC function is not possible without coordinated and directed long-distance cell migration.

Functional DCs are also of special interest as promising tools for new anti-tumor therapies [6]. *Ex vivo* generated DCs have been tested as vaccines in anti-cancer therapies and were able to expand T cells specific for cancer antigens [7], however, only about 1% of injected DCs migrated successfully to the draining lymph node [7] rendering the approach very inefficient. Thus, unraveling the mechanisms underlying DC migration is not only of cell biological interest, but also crucial for the optimization of DC-based therapeutic approaches.

While DC migration on two-dimensional (2D) surfaces requires adhesive forces, migration of DCs in three-dimensional (3D) environments was shown to occur independent of integrins. Instead the amoeboid-like migratory mode observed in 3D mainly relies on rapid cycles of actin polymerization [8]. Efficient actin polymerization requires actin nucleators such as the ARP2/3 complex, which catalyzes the formation of branched actin networks [9]. In order to be catalytically active, ARP2/3 requires stimulation by nucleation promoting factors (NPFs) like WASP. NPFs in turn are controlled by small GTPases of the Rho family including Cdc42 and Rac, which release them from auto-inhibition [9]. While the consequences of ARP2/3 loss on DC migration have not been reported, depletion of Rac1/2 [10], Cdc42 [11], WASP [12] or the actin regulator Eps8 [13] severely impaired DC migration to lymph nodes. Dysfunction of WASP is in fact associated with the primary immunodeficiency disorder Wiskott-Aldrich syndrome, which comprises an increased susceptibility to severe and life-threatening infections [14], illustrating the importance of regulated actin dynamics for the proper functioning of the immune system. However, ARP2/3 is not only controlled by activators, but also by a number of inhibitory factors, yet the physiological relevance of ARP2/3 inhibitors on DC migration has not been addressed. We have previously identified the AP-1-associated adaptor protein Gadkin [15] as a direct interactor of ARP2/3 [16]. In B16F1 melanoma cells, Gadkin sequestered ARP2/3 on endosomal vesicles in the absence of pro-migratory signaling thereby inhibiting ARP2/3-dependent processes such as cell spreading and cell migration [16].

Consistent with a role of Gadkin in DC physiology, we here report alterations in Gadkin protein levels and its posttranscriptional modification upon LPS-induced DC maturation. Capitalizing on a Gadkin-deficient mouse model previously established in our lab [16], we generated Gadkin-deficient bone marrow-derived DCs to examine the role of Gadkin in DC migration *in vitro* and *in vivo*. Loss of Gadkin led to an increase in polymerized actin levels and impaired DC motility in both two-dimensional (2D) and three-dimensional (3D) *in vitro* environments. However, *in vivo* migration of Gadkin-deficient DCs appeared to be unaffected, likely due to compensatory mechanisms and the known robustness of DC migration [8].

## Results

### Gadkin is expressed in immune cells

**Fig 1:**
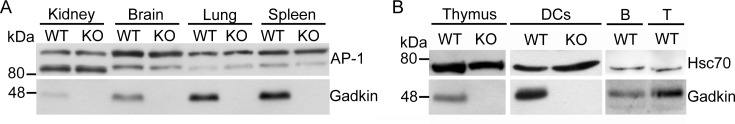
Gadkin is expressed in lymphoid organs and in immune cells. A. Lysates of WT and Gadkin^-/-^ (labeled as KO) tissues were analyzed by immunoblotting using Gadkin- and AP-1-specific antibodies. B. Lysates of WT and Gadkin^-/-^ thymus, of DCs derived from WT and Gadkin^-/-^ bone marrow, as well as of B lymphocytes (i.e. B220-positive B cells) and T lymphocytes (i.e. CD3-positive T cells) isolated from spleen were analyzed by immunoblotting using Gadkin-specific antibodies. Detection of the constitutively expressed molecular chaperone heat shock cognate protein 70 (Hsc70) was used as loading control. B, B-lymphocytes; T, T-lymphocytes.

The AP-1-associated adaptor protein Gadkin is present in a wide range of tissues [16]. The high expression level of Gadkin in the spleen (Fig 1A), an important secondary lymphoid organ, prompted us to determine the expression of Gadkin in the thymus as a primary lymphoid organ and in several important subsets of immune cells. Interestingly, Gadkin was not only detected in thymus, but also in primary bone marrow-derived DCs (BMDCs), and in B and T lymphocytes isolated from spleen (Fig 1B). Parallel analysis of Gadkin-deficient tissues verified the specificity of the bands obtained by immunoblotting with human [17] resp. newly generated mouse Gadkin-specific antibodies (for details about antibodies see Table 1). These results demonstrate the expression of the ARP2/3 inhibitor Gadkin in highly motile immune cells, including BMDCs, and thus lay the basis for investigating the role of Gadkin in DC migration.

**Table 1:** Antibodies.

Antigen	Species	Clone name or catalog #	Supplier	IF	WB	FACS	Comments
Actin	mouse	ac-15	Sigma-Aldrich		1:1000		
AP-1	mouse	610386	BD Biosciences	1:100			specific for γ-adaptin
B220/CD45R	rat	RA3-6B2	BD Pharmingen			1:100	biotin-labeled
CD3e	hamster	145-2C11	eBioscience			1:100	FITC-labeled
CD8a	rat	53–6.7	eBioscience			1:100	APC-labeled
CD11c	hamster	N418	Invitrogen			1:400	FITC-labeled
CD40	rat	1C10	eBioscience			1:200	APC-labeled
CD80	hamster	16-10A1	eBioscience			1:100	PE-labeled
CD86	rat	GL1	eBioscience			1:100	APC-labeled
CD197/CCR7	rat	4B12	eBioscience			1:10	APC-labeled
Gadkin	rabbit	#13	home-made		1:500		anti-human Gadkin; purified [17]
Gadkin	rabbit	854-F2	home-made	1:50	1:5000		anti-mouse Gadkin; purified
Hsc70	mouse	MA3006	Affinity Bioreagents		1:5000		
p34	rabbit	07–227	Millipore	1:400			

IF, immunofluorescence; WB, western blotting.

### Gadkin is not required for DC differentiation and maturation

**Fig 2:**
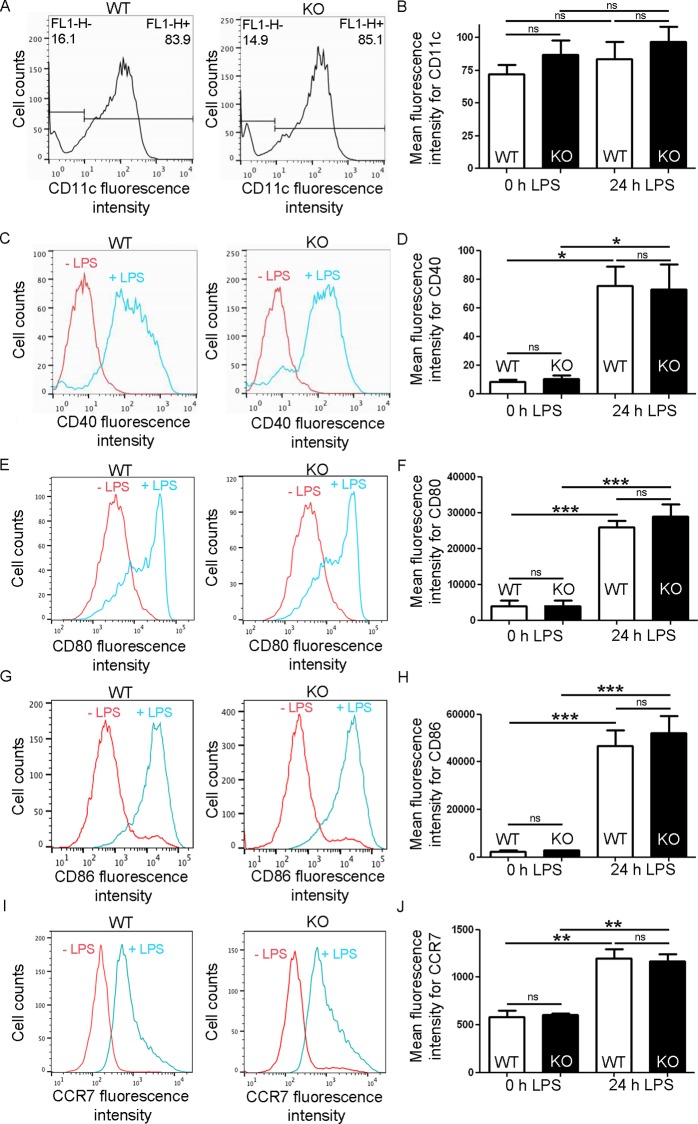
Loss of Gadkin does not affect *in vitro* DC differentiation and maturation. Bone marrow-derived WT and Gadkin^-/-^ DCs both display hallmark features of DC differentiation and maturation. A. Untreated, immature WT and Gadkin^-/-^ DCs were analyzed by flow cytometry with CD11c-specific fluorescently labeled antibodies. The diagrams depict the number of cells counted at different fluorescence intensities. Cells with a fluorescence intensity of at least 10^1^ were defined as CD11c-positive amounting to 84–85% of all cells. B. Quantification of mean fluorescence intensity for CD11c in immature and 24 h LPS-matured WT and Gadkin^-/-^ BMDCs based on flow cytometry (N = 3 independent experiments). C,E,G,I. Immature WT and Gadkin^-/-^ BMDCs were left untreated (red line) or were matured by incubation with LPS for 24 h (blue line) and subsequently analyzed by flow cytometry with the indicated fluorescently labeled antibodies. The diagrams depict the number of cells counted at different fluorescence intensities for both conditions illustrating the increase in surface expression of the tested markers after LPS treatment. D,F,H,J. Quantification of mean fluorescence intensity for the indicated marker proteins in immature and LPS-matured (24 h) treated WT and Gadkin^-/-^ BMDCs based on flow cytometry (N = 3 independent experiments for D and J, N = 4 independent experiments for F and H, significance was tested with one-way ANOVA followed by Tukey´s test to compare all groups, * = p<0.05, ** = p<0.01, *** = p<0.001).

Immature BMDCs were differentiated *in vitro* by culturing bone marrow suspensions of WT and Gadkin^-/-^ mice in the presence of granulocyte macrophage colony-stimulating factor (GM-CSF). Successful differentiation was confirmed by flow cytometrical analysis of CD11c surface levels, a marker protein of mouse DCs. WT and Gadkin^-/-^ BMDCs expressed CD11c to a similar degree (Fig 2A and 2B) indicating that BMDC differentiation is Gadkin-independent.

After eight days of culture, BMDC maturation was induced by treatment with bacterial lipopolysaccharide (LPS), a Toll-like receptor ligand, which triggers upregulation of a number of molecules that are critical for the function of mature DCs, among them major histocompatibility complex II (MHCII), the chemokine receptor CCR7 and co-stimulatory molecules such as CD40, CD80 and CD86. To assess DC maturation we measured surface levels of CD40, CD80, CD86 and CCR7, which are common markers to distinguish between immature and mature DCs in mice [18]. As expected, immature BMDCs expressed low levels of these markers, while LPS-treated BMDCs displayed strongly and significantly elevated levels of all markers (Fig 2C–2J). There was no difference in the surface levels of the tested markers between WT and Gadkin^-/-^ BMDCs indicating that *in vitro* DC maturation is not affected by loss of Gadkin.

**Fig 3:**
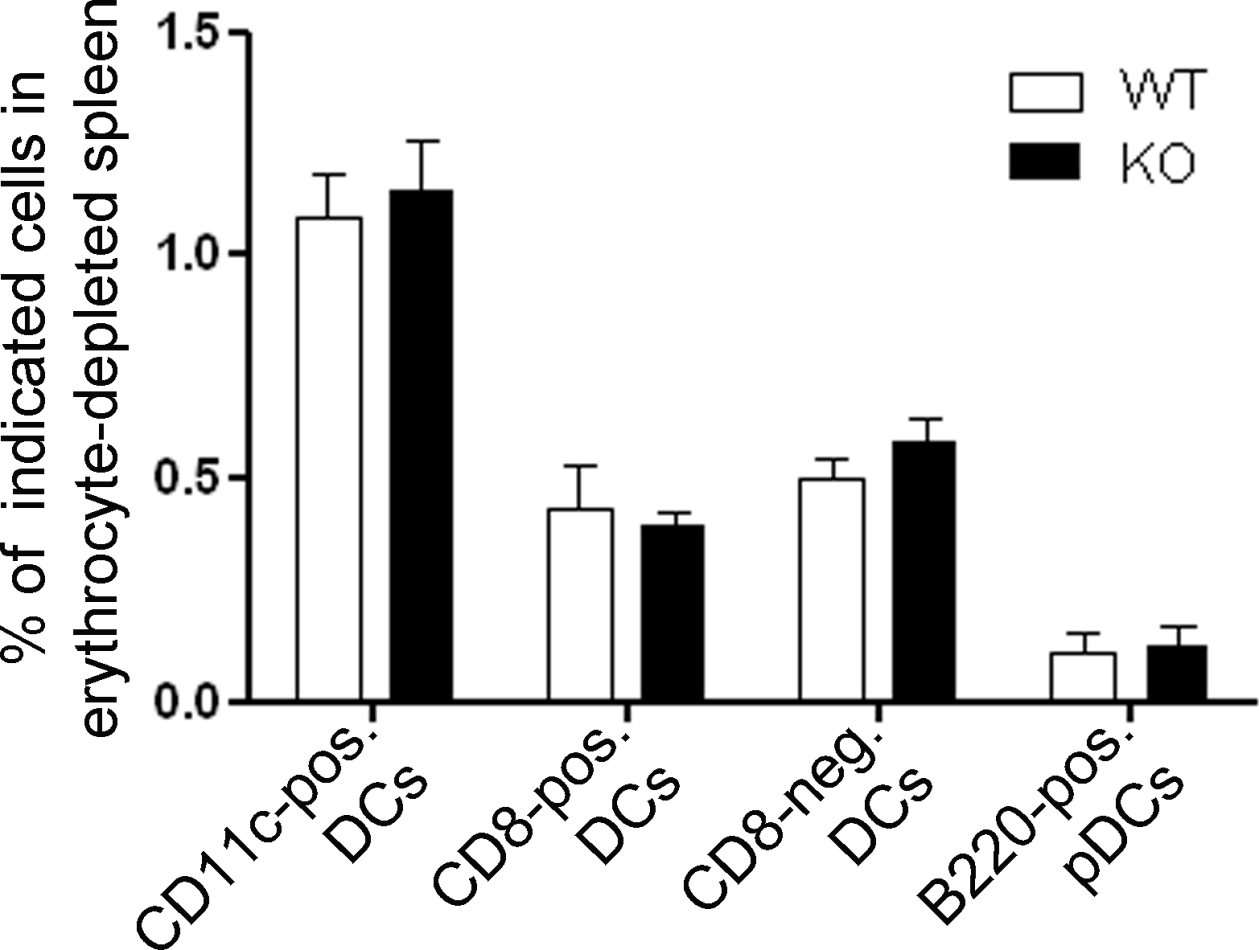
Loss of Gadkin does not affect generation of splenic DC subsets *in vivo*. WT and Gadkin^-/-^ spleens were processed into erythrocyte-depleted cell suspensions, and the percentage of different DC subsets relative to total splenocytes was quantified by flow cytometry using CD11c, CD8 and B220 as markers for the different DC populations (N = 3 independent experiments). Pos, positive; neg, negative; pDCs, plasmacytoid DCs.

To analyze whether this holds true for *in vivo* DC differentiation we quantified the percentage of different DC subsets in erythrocyte-depleted cell suspensions derived from WT and Gadkin^-/-^ spleens by flow cytometry. First we compared the overall abundance of DCs as characterized by expressing CD11c between WT and Gadkin^-/-^ spleens. There was neither a difference in the percentage of CD11c-positive cells relative to the entire splenocyte population (Fig 3) nor a change in the absolute number of spleen-resident CD11c-positive cells (absolute number in KO as % of WT: 108.5 ± 10.7 (N = 2)) between WT and Gadkin^-/-^ mice. We then went on to evaluate different DC subsets since DCs form a very heterogeneous group of cells. The main populations present in murine spleen are conventional DCs, which are subdivided in CD8-positive DCs, the most efficient subset for cross-presentation, and CD8-negative DCs, the more potent CD4+ T cell activators, as well as B220-positive plasmacytoid DCs, which are especially efficient in secreting anti-viral cytokines [19]. However, when quantifying these different DC subsets, we also did not detect any changes between WT and Gadkin^-/-^ spleens (Fig 3). Thus DCs arise *in vivo* with the same efficiency in WT and Gadkin^-/-^ mice arguing against a differentiation defect.

### LPS-induced BMDC maturation regulates Gadkin protein levels and posttranslational modification

**Fig 4:**
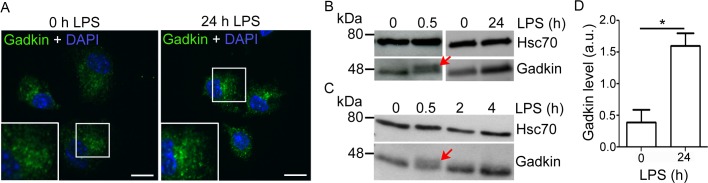
Gadkin is regulated in an LPS-dependent manner. A-D. LPS treatment of BMDCs increases Gadkin levels and induces its transient posttranscriptional modification. A. Immature WT BMDCs were either left untreated or incubated for 24 h with LPS. Subsequently, cells were allowed to spread on fibronectin-coated cover slips for 15 min to achieve a more uniform morphology prior to analysis. Cells were processed for immunofluorescence and labeled with Gadkin-specific antibodies. Nuclei were stained with DAPI. Insets show 2x enlarged perinuclear area. Scale bar: 10 μm. B,C. Lysates from WT BMDCs treated with LPS for the indicated times were analyzed by immunoblotting using Gadkin- and Hsc70-specific antibodies. The closely spaced upper band is indicated by red arrows. D. Quantification of immunoblot images like the one depicted in B. Gadkin levels were normalized to Hsc70 levels derived from the same blot. (N = 3 independent experiments, unpaired two-tailed t-test; * = p<0.05; a.u., arbitrary units).

The maturation of DCs in response to pathogens involves a plethora of changes that prime DCs for migration to lymph nodes and subsequent T cell activation. If Gadkin is an important factor for these processes, it might be regulated upon LPS treatment. In fact, when we analyzed Gadkin protein levels 24 h post LPS treatment by immunofluorescence and immunoblotting, we detected a >2-fold significant increase in its expression level, while Gadkin´s subcellular localization to the perinuclear recycling compartment remained unaltered (Fig 4A, 4B and 4D). In addition to the maturation-induced long-term increase in Gadkin levels, which occurs on the time scale of hours, we observed the appearance of a very closely spaced additional Gadkin-specific band of lower electrophoretic mobility in immunoblots. This second band appeared about 30 min after LPS application and had disappeared again 1.5 h later, indicative of a transient posttranslational modification of Gadkin (Fig 4B and 4C). While this is the first indication of Gadkin being regulated by posttranslational modification, the exact nature of this modification remains to be determined. The coordinated regulation of Gadkin in the context of DC maturation strongly argues for a role of Gadkin in DC function.

### Loss of Gadkin affects the actin cytoskeleton of BMDCs

**Fig 5:**
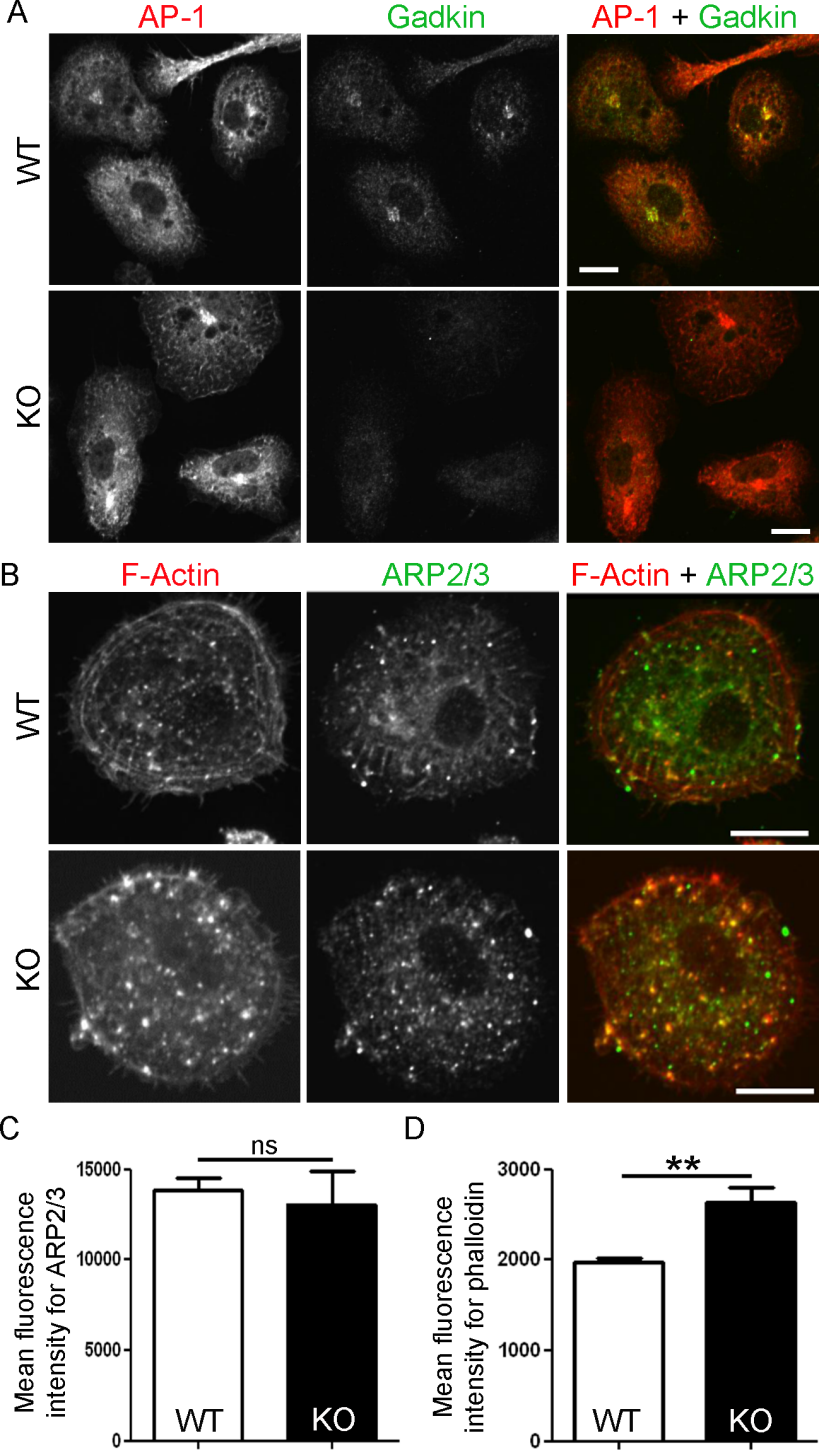
Loss of Gadkin leads to an increase of F-actin. A. AP-1 localization is not altered in the absence of Gadkin. WT and Gadkin^-/-^ BMDCs were matured for 24 h with LPS. Subsequently, BMDCs were allowed to spread on fibronectin-coated cover slips for 240 min prior to fixation and staining with Gadkin- and AP-1-specific antibodies. Scale bar: 10 μm. B. ARP2/3 and F-actin localization appears unaltered in the absence of Gadkin. BMDCs treated as in A. were incubated with ARP2/3-specific antibodies against the subunit p34/ARPC2 and with fluorescently labeled phalloidin to stain F-actin. Scale bar: 10 μm. C. The level of ARP2/3 is not changed in Gadkin^-/-^ BMDCs. Immunofluorescence-based quantification of the mean fluorescence intensity of the ARP2/3 subunit p34/ARPC2 (N = 5 independent experiments, unpaired t-test, ns = non significant). D. Gadkin^-/-^ BMDCs contain more F-actin. Flow cytometry-based quantification of phalloidin-stained suspension cells that had been starved for 1 h (N = 7 independent experiments, unpaired t-test, ** = p<0.01).

AP-1 and ARP2/3 are two of the most important interactors of Gadkin expressed by DCs. However loss of Gadkin did not change the intracellular distribution of AP-1 (Fig 5A) or ARP2/3 (Fig 5B). Likewise the amount of ARP2/3 quantified via its subunit p34/ARPC2 did not differ in Gadkin^-/-^ BMDCs (Fig 5C). Still, based on the hypothesis that Gadkin acts also in DCs as a negative regulator of ARP2/3, the amount of polymerized actin might be increased in Gadkin-deficient BMDCs. As Gadkin is known to sequester ARP2/3 especially in non-stimulated cells [16], we quantified the level of polymerized actin in serum-starved LPS-matured BMDCs by staining them with fluorescently-labeled phalloidin prior to flow cytometrical analysis. Indeed, mature Gadkin^-/-^ BMDCs displayed a significantly elevated level of F-actin, which is in line with our hypothesis (Fig 5D).

### Loss of Gadkin does not affect macropinocytosis of BMDCs

**Fig 6:**
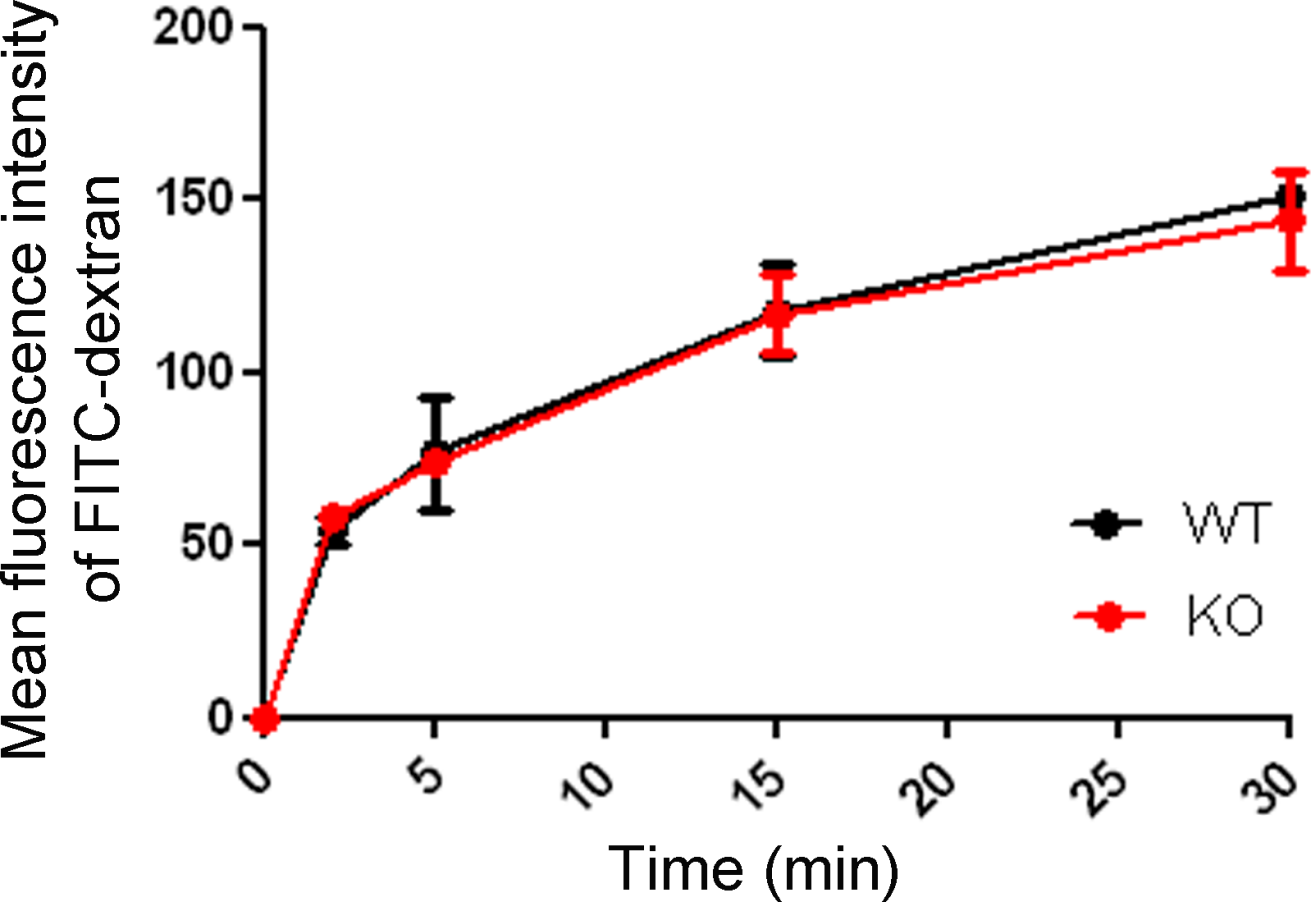
Macropinocytosis is unaltered in Gadkin^-/-^ BMDCs. Immature WT and Gadkin^-/-^ BMDCs were incubated with FITC-dextran for the indicated time intervals. FITC-Dextran uptake was quantified by flow cytometry (N = 2 independent experiments).

To address whether the observed difference in F-actin affects actin-dependent processes in BMDCs we started by investigating macropinocytosis of FITC-dextran. Macropinocytosis denotes the bulk uptake of fluid-phase solutes via the actin-dependent formation of large vesicles [20]. DCs are known to constitutively macropinocytose large quantities of exogenous solute as part of their sentinel function [20]. However, when comparing the amount of macropinocytosed FITC-dextran after different time intervals, we did not detect any difference between WT and Gadkin^-/-^ BMDCs (Fig 6).

### Loss of Gadkin does not affect cell spreading of BMDCs

**Fig 7:**
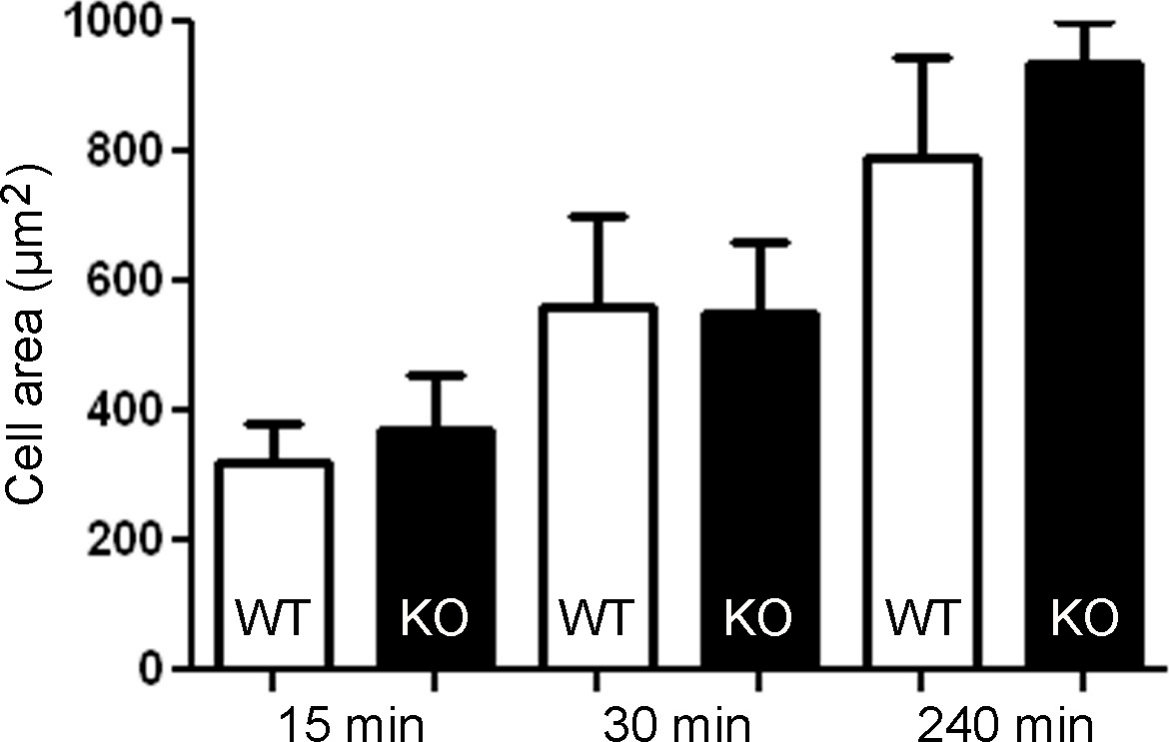
Gadkin is dispensable for spreading of mature BMDCs. A. Gadkin^-/-^ BMDCs display no alterations in cell spreading. WT and Gadkin^-/-^ BMDCs were matured for 24 h with LPS. Subsequently, cells were allowed to spread on fibronectin-coated cover slips for 15 to 240 min. Cells were processed for immunofluorescence and incubated with phalloidin to stain F-actin and thus visualize cell shape. Cell size was quantified at the indicated times post-plating (N = 2 independent experiments for 15 min time point and N = 4 for 30 and 240 min time points).

The ARP2/3 complex is not only implicated in macropinocytosis, but known to be required for efficient cell spreading [21]. Previously, we have shown that loss of Gadkin promotes B16F1 melanoma cell spreading [16]. To assess whether loss of Gadkin also affects spreading of mature BMDCs, WT and Gadkin^-/-^ BMDCs were plated onto fibronectin 24 h post LPS treatment and allowed to spread for different time intervals. However, the spreading behavior of Gadkin^-/-^ BMDCs was indistinguishable from that of WT cells (Fig 7).

### Loss of Gadkin impairs CCR7-driven DC migration in 2D

**Fig 8:**
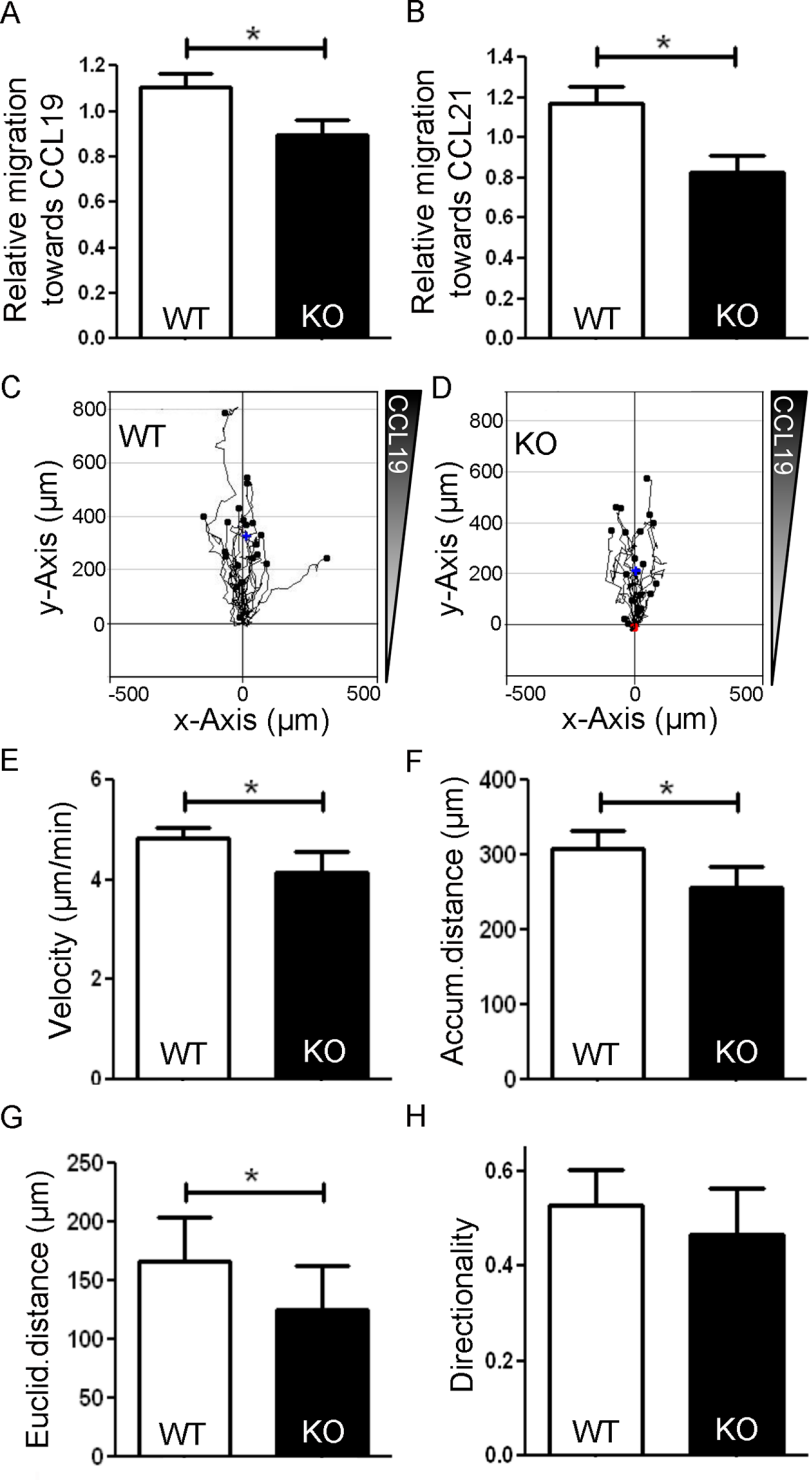
Loss of Gadkin impairs BMDC chemotaxis in 2D. A,B. LPS-matured WT and Gadkin^-/-^ BMDCs were seeded into Transwell inserts and allowed to migrate for 3 h towards 250 ng/ml CCL19 (A) resp. 250 ng/ml CCL21 (B). Migration towards the chemokine was analyzed by flow cytometry. Values were subtracted for unspecific migration towards chemokine-free medium to determine the specific migration towards chemokine and then normalized to the average value of each experiment (N = 6 independent experiments, unpaired two-tailed t-test, * = p<0.05). C-H. LPS-matured WT and Gadkin^-/-^ BMDCs were seeded into a 2D chemotaxis chamber. BMDCs were allowed to migrate for 4 h towards 650 ng/ml CCL19, and their migratory behavior was evaluated based on bright-field images obtained at 2 min intervals. C,D. Migration tracks of 21 WT (C) and 23 Gadkin^-/-^ BMDCs (D) centered on the same starting point. Center of mass depicted as blue cross (WT: x = 10.57 μm, y = 326.38 μm, KO: x = 4.26 μm, y = 213.79 μm). Velocity (E) was quantified as well as the accumulated (F) and Euclidian distance (G) covered by the BMDCs. Directionality (H) was calculated as Euclidian distance divided by accumulated distance (N = 4 independent experiments, at least 20 cells per experiment were tracked, paired two-tailed t-test for distances, paired one-tailed t-test for velocity, * = p<0.05).

The ARP2/3 complex is crucial for cellular motility, and depletion of ARP2/3 regulators in DCs impaired DC migration in 2D environments [22]. To determine whether loss of Gadkin likewise affects DC migration in 2D, we seeded LPS-matured WT and Gadkin^-/-^ BMDCs into the upper reservoirs of Transwells and let them migrate for 3 h towards the chemokine CCL19 or CCL21 present in the lower reservoirs. Subsequently, the number of cells having migrated to the lower reservoir was assessed by flow cytometry. For both chemokines tested Gadkin^-/-^ BMDCs migrated significantly less efficiently towards the chemokine than WT DCs (Fig 8A and 8B). This migration impairment may either indicate a defective response to the chemotactic cue or a mechanistic problem with cell migration itself, possibly due to alterations in actin dynamics. The latter possibility was supported by the fact that Gadkin^-/-^ BMDCs displayed impaired random migration in the absence of chemokines (WT: 1.15 ± 0.07, KO: 0.85 ± 0.07, p<0.05 in paired one-tailed t-test, N = 6 independent experiments). Differences in DC migration might be caused by alterations of DC velocity or in their directional persistence. As the Transwell assay is an endpoint assay, which does not allow to dissect these different migration parameters, we turned to live cell imaging of DCs in 2D chemotaxis chambers, where DCs migrate along a chemokine gradient. Analysis of DC tracks revealed a moderate, but significant decrease in the velocity of LPS-matured Gadkin^-/-^ BMDCs consistent with a significantly reduced accumulated and Euclidian distance covered by the Gadkin^-/-^ BMDCs in the course of the experiment, while the directionality of migration was not altered (Fig 8C–8H).

### Loss of Gadkin affects DC migration in 3D environments

**Fig 9:**
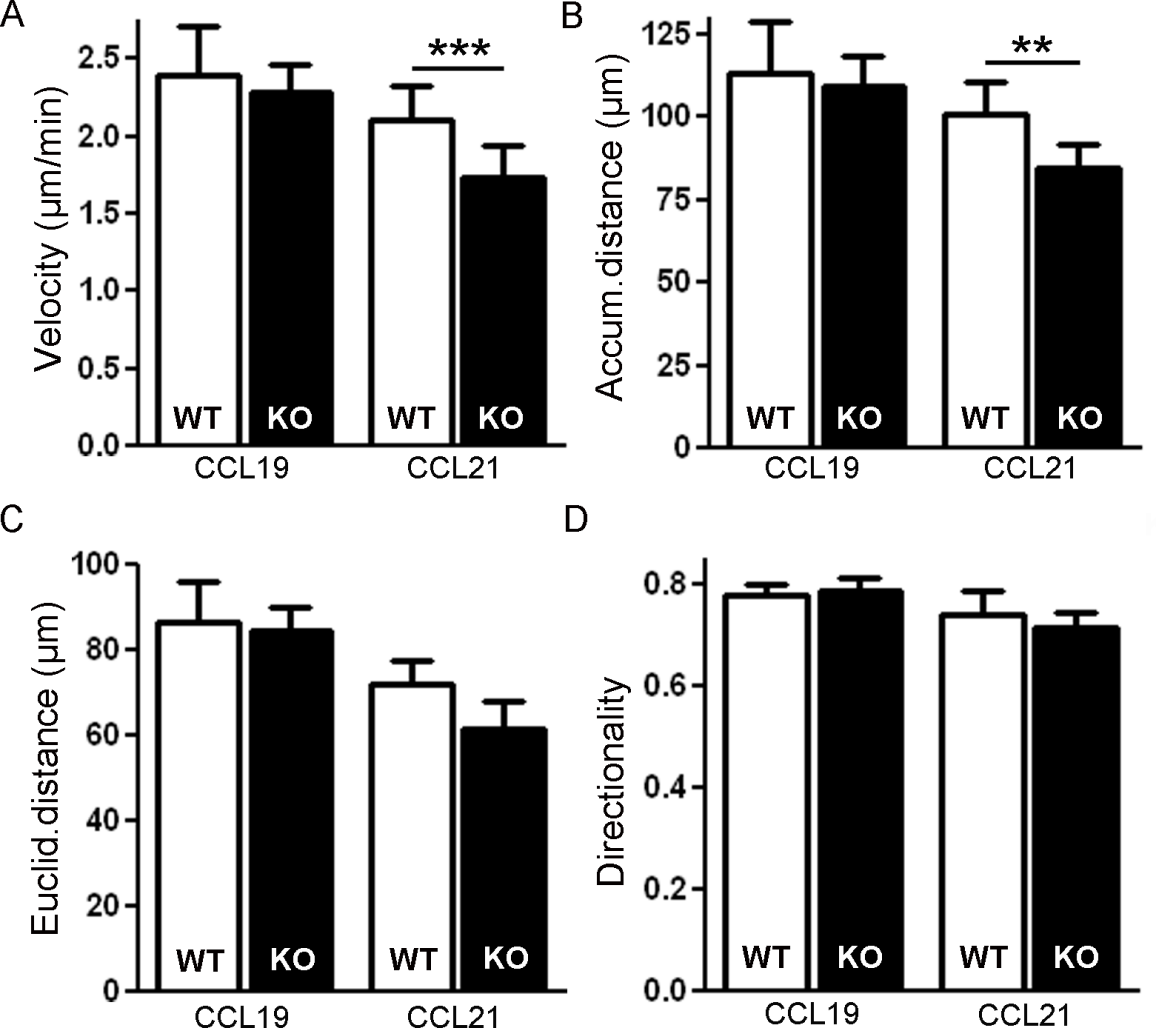
Loss of Gadkin impairs directed BMDC migration towards CCL21 gradients in 3D collagen gels. A-D. WT and Gadkin^-/-^ BMDCs matured in the presence of LPS for 24 h were embedded into 1.6 mg/ml collagen gels. The upper gel surface was covered with medium containing 650 ng/ml CCL19 or 650 ng/ml CCL21, respectively. BMDC chemotaxis was monitored by bright-field real-time microscopy for 6 h. Images were obtained at 4 min intervals. Velocity of migrating cells (A) was quantified as well as the accumulated (B) and Euclidian distance (C) covered by the BMDCs. Directionality (D) was calculated as Euclidian distance divided by accumulated distance (N = 5 independent experiments, at least 20 cells were tracked per experiment, paired two-tailed t-test for comparison of WT and Gadkin^-/-^ BMDCs subjected to same condition, *** = p<0.001; ** = p<0.01).

DC migration in 2D and 3D environments follows different modes. 2D migration of DCs requires adhesive forces to generate traction, while their migration in 3D environments can solely be driven by the force of actin-network expansion [8, 22]. In line with these mechanistic differences, ablation of genes that regulate actin dynamics like Cdc42 caused especially pronounced defects in 3D DC migration [11]. To determine whether loss of Gadkin also has a greater impact on 3D migration, we embedded BMDCs into collagen type I gels mimicking collagen-rich connective tissue and therefore constituting an *in vivo* like environment [22]. LPS-matured WT and Gadkin^-/-^ BMDCs seeded into a chemokine-free collagen gel moved with comparable speed (WT: 2.03±0.21 μm/min, N = 3; KO: 2.11±0.15 μm/min, N = 4) and similar directionality (WT: 0.60±0.02, N = 3; KO: 0.61±0.01, N = 4). The migratory behavior of Gadkin^-/-^ BMDCs was likewise unaltered in the presence of a CCL19 gradient (Fig 9A–9D). Interestingly, less efficient 3D migration of Gadkin^-/-^ BMDCs was only observed in the presence of CCL21 (Fig 9A–9D), the chemokine towards which migration of Gadkin^-/-^ BMDCs had also been more severely impaired in the Transwell assay and which is *in vivo* the more important chemokine for DC homing [5]. Gadkin^-/-^ BMDCs migrated significantly slower than WT BMDCs along the CCL21 gradient, while their directionality was again unaffected. In summary, loss of Gadkin causes moderate but significant defects in 3D migration in the presence of CCL21, which are in the same range as defects observed during 2D *in vitro* chemotaxis.

### Loss of Gadkin can be compensated *in vivo* resulting in unaltered DC homing to draining lymph nodes

Collagen gels represent a simplified approximation of the complex fibrillar environment of dermal interstitial tissues and cannot recapitulate all migration steps, which DCs have to achieve on their way from the dermal interstitium via afferent lymphatics to the draining lymph node. Therefore, we turned to *in vivo* migration experiments to elucidate whether the *in vitro* migration impairment of Gadkin^-/-^ BMDCs translates into a decreased arrival at draining lymph nodes *in vivo*. The disturbed chemotaxis of Gadkin^-/-^ BMDCs towards CCL21 might be especially detrimental *in vivo*, as endogenous gradients of CCL21 within mouse skin were shown to guide DCs towards lymphatic vessels [4].

**Fig 10:**
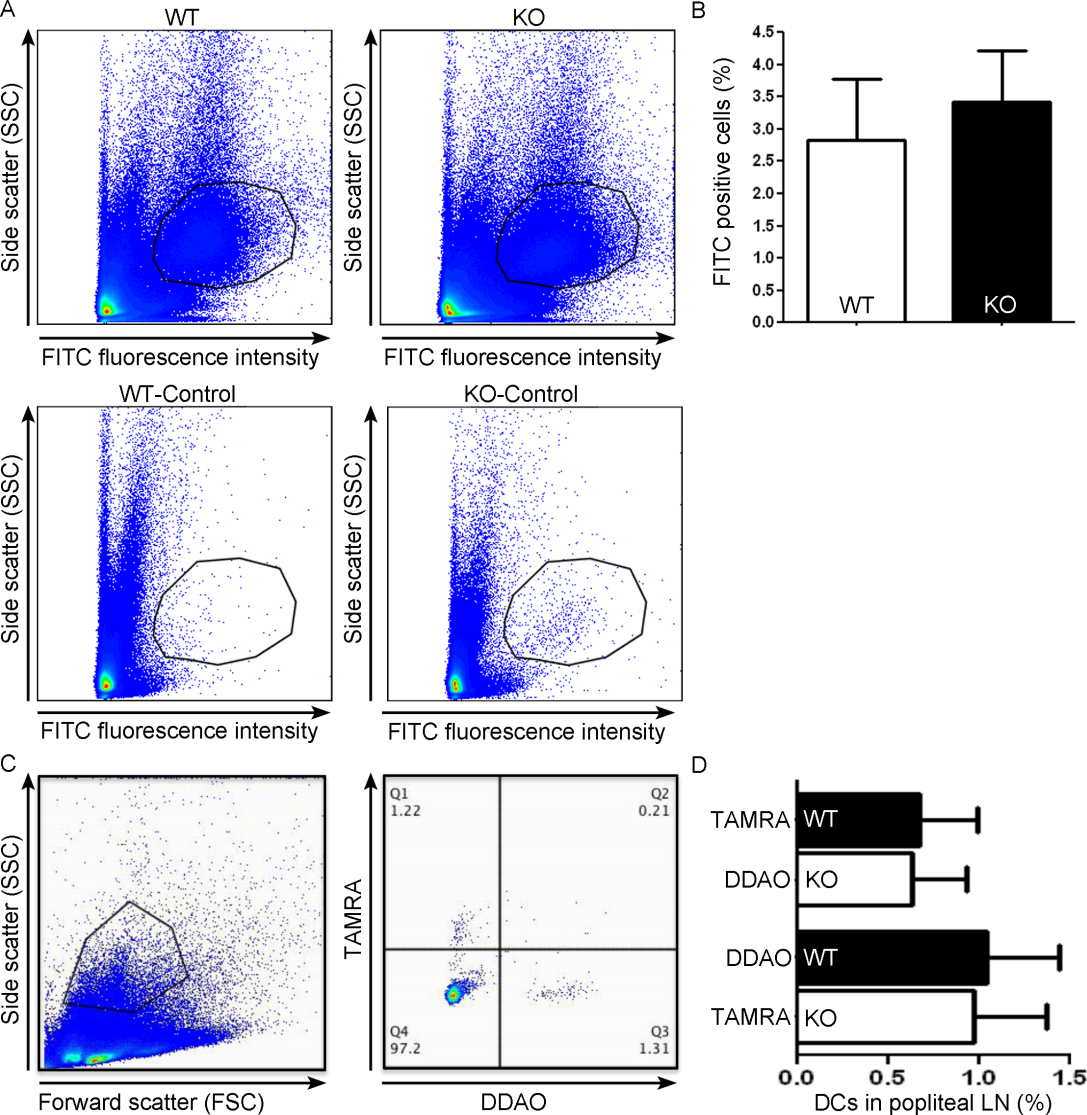
Unaltered *in vivo* DC migration in absence of Gadkin. A,B. Skin irritant-induced DC migration to lymph nodes is not altered in the absence of Gadkin. FITC in acetone/dibutylphtalate was painted onto the shaved skin of WT and Gadkin^-/-^ mice. The percentage of FITC-positive DCs migrating to the draining inguinal lymph nodes was assessed by flow cytometry. A. Representative examples of gating to select FITC-positive DCs for analysis (top). Lower panels show control lymph nodes devoid of FITC-labeled DCs. B. Quantification of the percentage of FITC-positive cells in draining lymph nodes (N = 5 mice per genotype from 2 independent experiments). C,D. Labeled LPS-matured WT and Gadkin^-/-^ BMDCs reach the draining popliteal lymph nodes with comparable efficiency after injection into foot pads. C. Representative example of gating to select transferred TAMRA- resp. DDAO-positive BMDCs for analysis. D. Quantification of the percentage of TAMRA- and DDAO-positive WT and Gadkin^-/-^ BMDCs in popliteal lymph nodes (LN) (N = 3 mice per genotype from 2 independent experiments).

We assessed *in vivo* migration by two approaches. First we analyzed migration of endogeneous skin-resident DCs in response to skin irritation. To this end, we cutaniously applied the skin irritant dibutylphthalate together with the dye FITC to the abdomen of WT and Gadkin^-/-^ mice. Dibutylphthalate induces DC maturation and thereby triggers their migration to draining lymph nodes, while FITC is efficiently taken up by skin-resident DCs resulting in their fluorescent labeling. Arrival of FITC-positive DCs in the draining lymph node was assessed by flow cytometry and revealed no significant difference between WT and Gadkin^-/-^ DC migration (Fig 10A and 10B). In a second approach we labeled *in vitro* differentiated and matured WT and Gadkin^-/-^ BMDCs with different dyes and injected them in a 1:1 mixture into the footpads of C57BL/6 mice. Similar numbers of transferred WT and Gadkin^-/-^ BMDCs arrived in the draining popliteal lymph node, as determined by flow cytometry (Fig 10C and 10D). In conclusion, loss of Gadkin does not detectably impair the efficiency of DC migration from dermal tissue to draining lymph nodes *in vivo*, suggesting that loss of Gadkin in DCs can be compensated, presumably by changing the mode of migration.

## Discussion

Immune cell migration throughout the organism is essential for successful immune surveillance and the induction of innate and adaptive immunity. This particularly holds true for DCs, which have to navigate throughout the body in order to sample foreign antigens in peripheral tissues and display them to T cells in secondary lymphoid organs to mount immune responses.

DC migration relies on the dynamic remodeling of the actin cytoskeleton in response to extracellular cues, for which ARP2/3 is a crucial factor. We have previously demonstrated that Gadkin functions as an ARP2/3 inhibitor and that loss of Gadkin facilitates ARP2/3-dependent cell spreading and cell migration in melanoma cells. Here, we addressed whether Gadkin deficiency also alters migration of DCs. Our data show that loss of Gadkin significantly increases F-actin levels and moderately, but likewise significantly impairs BMDC migration *in vitro*. These findings are in contrast to our initial hypothesis, derived from experiments with B16F1 melanoma cells, that migration might be facilitated in the absence of Gadkin. However, actin polymerization is controlled by a plethora of regulatory factors, and the cell-type specific composition of the actin regulatory proteome might modulate the precise effect of a single regulatory protein on cell migration. The fact that loss of Gadkin affects 2D migration also in the absence of chemokines is consistent with a direct effect of Gadkin on actin dynamics by ARP2/3 inhibition.

However, in 3D collagen gels Gadkin^-/-^ BMDCs were only significantly slower in response to a CCL21 gradient. This suggests that Gadkin does not affect actin-based cell motility in the 3D migration mode, but rather modulates chemotactic sensing. However, Ca^2+^ levels did not appear to be different between LPS-matured WT and Gadkin^-/-^ BMDCs following chemokine application (signal intensity in KO as % of WT upon CCL19: 92.9 ± 7.0 (N = 6); upon CCL21: 108.9 ± 8.1 (N = 5)). Due to its role in recycling endosomal membrane traffic Gadkin might putatively affect the composition of the DC surface proteome [17] thereby modulating DC sensitivity to chemotactic cues. However, as CCL19 and CCL21 are both ligands for the same chemotactic receptor, CCR7, this scenario seems unlikely, especially as we did not observe any difference in the surface level of CCR7 (Fig 2J). Whereas both chemokines bind CCR7 with similar affinity [23], CCL19 and CCL21 elicit different outcomes upon receptor activation in regards to CCR7 internalization [24], downstream signaling events [25, 26] and migratory mode [27]. In fact, CCL21, but not CCL19 activates DC adhesion to ligands like ICAM-1 [27]. Because of its link to endosomal trafficking and actin dynamics Gadkin might be involved in the regulation of the CCL21-dependent adhesion thereby affecting migration efficiency. Alternatively, Gadkin could modulate CCL21-mediated intracellular CCR7 trafficking and signaling responses, hypotheses which await further testing.

In contrast to our *in vitro* results *in vivo* DC migration appeared not to be affected by the loss of Gadkin limiting the physiological significance of the observed *in vitro* defects. This highlights the importance of *in vivo* experiments to test the physiological relevance of *in vitro* data and, secondly, it suggests a high plasticity of the migration process as well as potential compensatory changes in the constitutive Gadkin KO animals that allow overall DC migration to still work in the face of minor mechanistic defects. This is in line with previous observations on the robustness of leukocyte migration. Even upon complete abrogation of adhesion and contraction or severe interference with actin dynamics, leukocytes were reported to still be able to move [8, 22]. Depending on the chemokine being present and on the way it is presented within the tissue (i.e. soluble vs. surface-bound) DCs can switch between distinct migratory modes that involve different degrees of surface adhesion [23]. This ability to alternate between different migration modes might well mask effects of Gadkin that are specific for one of the migration modes, especially as DCs encounter *in vivo* a mixture of differently presented chemokines, presumably causing a greater flexibility in their migration pattern than in our *in vitro* experiments where only one chemokine was present at a time.

The notion that the plasticity of DC migration might obscure effects of Gadkin deficiency is especially appealing, as different facts point to an involvement of Gadkin in DC physiology. We have shown that Gadkin is not only expressed by BMDCs, but that its protein levels are upregulated upon LPS-induced DC maturation suggesting a specific function for Gadkin in mature DCs. In addition, we detected for the first time a post-translational modification of Gadkin, which transiently occurs shortly after the induction of DC maturation by LPS. The exposure of DCs to inflammatory stimuli like LPS was reported to first induce a brief interval of immobility and enhanced endocytic antigen uptake, before DCs re-arrange their repertoire of chemokine receptors and migrate to lymph nodes [1, 28]. The transient post-translational modification of Gadkin coincides with the described acute events after LPS encounter, thus suggesting a role for Gadkin in the early phase of DC maturation, potentially related to the transient stalling of migration.

Furthermore, Gadkin is a candidate modifier gene for colitis severity. The spontaneous ulcerative colitis (TRUC) developing in a DC-dependent manner in T-bet/RAG2 double knockout mice [29] is highly penetrant in BALB/c mice, but less severe in C57BL/6 mice [30]. The cytokine deficiency-induced colitis susceptibility-1 (cdcs1) locus, which encodes 21 genes expressed in DCs, was shown to control colitis severity. Gadkin, displaying 3.6-fold higher transcript levels, is one of the three cdcs1-encoded genes, which were shown to be upregulated in the more susceptible mouse strain [30]. This suggests that low Gadkin levels might be beneficial for colitis prevention and, in accordance with our results, strengthens the link between Gadkin and immune system functions. However, additional studies are clearly needed to elucidate the role of Gadkin in immunity and to identify its potential function in DC physiology.

## Materials and Methods

Secondary antibodies were either conjugated to horseradish peroxidase (HRP) for Western blot detection (GE Healthcare) or to Alexa-fluorophores in case of immunofluorescence experiments (Invitrogen).

### Animals

The generation of Gadkin^-/-^ mice is described in [16]. Animals were backcrossed onto a C57BL/6J background and housed on a 12 h light/dark cycle with food and water available *ad libitum* in a specific pathogen free (SPF) facility. All experiments in the present study were carried out in strict accordance with the guidelines of the Landesamt für Gesundheit und Soziales (LAGeSo) Berlin. The study was specifically approved by the ethics committee of the LAGeSo Berlin under animal experimentation permit number G 0185/14, and all efforts were made to minimize suffering. All treatments were performed under isoflurane anesthesia. Animals were sacrificed by cervical dislocation.

### Isolation of immune cell populations

Mouse spleens were dissected into small pieces, digested for 40 min in 10 mg/ml Collagenase D (Roche) at 37°C and mechanically dissociated using cell strainers (BD). Cells were washed in PBS, exposed to ice-cold red cell removal buffer (RCRB: 0.15 M NH_4_Cl, 0.1 mM EDTA in H_2_O) for 2 min to deplete erythrocytes, and washed twice in PBS before antibody staining was performed as described below. Splenic T and B lymphocytes were isolated from total splenocytes by FACS using anti-CD3e and anti-B220 surface staining, respectively. Cell sorting was performed on a BD FACS Aria III.

### Quantification of splenic DC subsets

Mouse spleens were dissociated into cell suspensions and depleted of erythrocytes as described above. Different DC subsets were quantified by flow cytometry after antibody staining against CD11c (for all splenic DCs), CD8 (to distinguish CD8-positive conventional DCs from CD8-negative ones) and B220 (to label plasmacytoid DCs). For the determination of absolute cell numbers 1x10^5^ microbeads (Life Technologies, F8887) were added to each sample before performing the staining protocol described below. After staining, the number of beads and cells within each sample was quantified by flow cytometry. Initial total numbers of a specific cell population were calculated as: initial number of cells = (10^5^ x number of cells measured) / number of beads measured.

### Generation of bone marrow-derived dendritic cells (BMDCs)

Ca. 6–9 weeks-old mice were sacrificed by cervical dislocation. Abdomen and hind legs were sterilized with 70% ethanol. Murine bone marrow was isolated from femur and tibiae of the hind legs by flushing the bones with ice-cold, sterile PBS using a 25G needle. Red blood cells were lysed in RCRB. The resulting cell suspension was applied to a 70 μm cell strainer and then centrifuged for 5 min at 300 x g at room temperature. 4x10^6^ cells were resuspended in 10 ml BMDC medium (RPMI 1640 containing 300 mg/l L-glutamine (ThermoFisher #11875), supplemented with 10% fetal bovine serum (life technologies #10270106), 55 μM β-mercaptoethanol (ThermoFisher #21985–023), 50 U/ml penicillin, 50 U/ml streptamycin (life technologies #15140) and 30 ng/ml recombinant mouse GM-CSF (affymetrix eBioscience #BMS325) and seeded into a 10 cm dish. On day 3, 6 and 8 GM-CSF-containing RPMI was renewed. Differentiated BMDCs were used for experiments on day 9.

### Preparation of lysates and immunoblot-based analysis

BMDCs were collected from the plate and centrifuged at 300 x g for 5 min at room temperature. The cell pellet was washed twice with PBS before lysis in lysis buffer (20 mM HEPES pH 7.4, 100 mM KCl, 2 mM MgCl_2_, 1% Triton X-100, 2 mM PMSF, 0.6% protease inhibitor cocktail (Sigma)). Adherent cell types were washed briefly with PBS on the plate before scraping into lysis buffer. After 5 min on ice lysates were centrifuged at 4°C for 5 min at 17000 x g. The protein concentration of the supernatant was determined by Bradford assay. Tissue extracts were obtained by tissue homogenization in homogenization buffer (320 mM sucrose, 4 mM HEPES pH 7.4, 2 mM PMSF, 0.3% protease inhibitor cocktail (Sigma)) using a potter (15 strokes at 1000 rpm). After centrifugation with 1000 x g at 4°C for 10 min, the supernatant was collected and again cleared by centrifugation for 5 min with 17000 x g at 4°C to yield the final tissue extract. Prior to immunoblotting lysates were adjusted to 1x Laemmli sample buffer. Samples were analyzed by SDS-PAGE and immunoblotting. Bound primary antibodies were detected by incubation with secondary antibodies conjugated to HRP and a chemiluminescent substrate. Signals were either detected on film or using an imaging system from Biorad for quantitative purposes. Band intensities were quantified with ImageJ and were normalized to Hsc70.

### Flow cytometry-based assays

Flow cytometry was used to quantify protein surface levels as well as polymerized actin and Ca^2+^ levels. For surface stainings, cells were blocked with 5% BSA in PBS for 20 min and subsequently washed with PBS. Antibodies were applied for 30 min at 4°C in FACS buffer (2% FCS, 10 mM EDTA in PBS in case of CD11c and CD40 stainings; 0.1% BSA, 0.05% NaN_3_ in PBS for all other stainings). For the detection of polymerized actin by intracellular phalloidin staining, BMDCs were starved for 1 h in serum-free BMDC medium before fixation with 4% paraformaldehyde. Fixed cells were permeabilized with 0.05% Triton X-100, 2% BSA in PBS for 30 min and stained with phalloidin-AF488 (1:50) for 1 h. After antibody staining all cells were washed twice with PBS before resuspension in FACS buffer. Samples were immediately analyzed with a FACSCalibur or FACSCanto (BD Biosciences) using CellQuest Pro and FACSDiva, respectively. Analysis of Ca^2+^ levels following cytokine application was performed by adjusting BMDCs to 1x10^6^ cells/ml and equilibrating them to Ca^2+^ flux buffer (145 mM NaCl, 5 mM KCl, 1 mM MgCl, 1 mM CaCl, 1 mM Na_2_HPO_4_, 5 mM Glucose, 10 mM HEPES pH 7.5) containing 4 μM Fluo-4 (life technologies) for 40 min at 37°C. Cells were washed once with flux buffer and resuspended in 500 μl flux buffer. Baseline fluorescence in the FITC channel was recorded for 60 s on a FACSCanto (BD). CCL19 or CCL21 were added (250 ng/ml), and FITC-fluorescence was again recorded for 120 s. Finally, 5 μM ionomycine were added to mobilize the entire Ca^2+^ pool, and FITC-fluorescence was measured for another 60 s. Data were analyzed with FlowJo V10.

### FITC-dextran uptake

BMDCs were carefully detached with a cell scraper and resuspended at 5x10^5^ cells per tube in full BMDC medium. Cells were stimulated for 30 min with 100 ng/ml LPS at 37°C and then incubated with 1 mg/ml FITC-dextran (life technologies) at 37°C for different durations. Dextran uptake was stopped by washing cells twice with ice-cold RPMI. Control samples were incubated with 1 mg/ml FITC-dextran, but kept on ice during the time course to determine FITC-dextran background surface binding levels. Stopped samples were kept in 2% goat serum in PBS on ice and rapidly analyzed by flow cytometry.

### Immunofluorescence

Mature or immature BMDCs were harvested and seeded onto 50 μg/ml fibronectin-coated coverslips. For immunofluorescence stainings cells were fixed with 4% PFA in PBS for 10 min at room temperature. Cells were permeabilized and blocked with 0.5% Triton X-100 and 10% goat serum in PBS (GSDB) for 5 min. Afterwards, samples were incubated with different primary antibodies in GSDB for 30 min at room temperature. After three short PBS washes, coverslips were incubated with species-specific Alexa488- or Alexa568-labeled secondary antibodies (Invitrogen) at a 1:200 dilution in GSDB for 1 h in the dark at room temperature. To visualize F-actin, Alexa568-coupled phalloidin (Invitrogen) was applied at a 1:50 dilution in GSDB together with the secondary antibodies. After the incubation, coverslips were washed three times with PBS and mounted onto microscopy slides with ImmuMount mounting solution (Thermo Electron) supplemented with 1 μg/ml DAPI to stain nuclei. Images were taken by spinning disc confocal microscopy using a Zeiss Axiovert 200M microscope (Carl Zeiss, Jena, Germany) operated by Volocity, equipped with an EM-CCD-camera and a View ERS Rapid Confocal Imager (Perkin Elmer, London, England). Image analysis was performed with Fiji, an ImageJ 1.47g package.

### 2D DC chemotaxis assay

BMDCs were stimulated with 200 ng/ml LPS for 24 h. Mature BMDCs were harvested and left to spread for at least 30 min in a chemotaxis chamber (μ-slice 3D chemotaxis chamber, Ibidi). A gradient of 650 ng/ml CCL19 was applied as described in the user manual. Time-lapse movies of migrating cells were acquired with a 10x objective on a Nikon Eclipse Ti epifluorescent microscope, equipped with an Andor sCMOS camera, an Okolab incubator for life cell imaging (set to 37°C and 5% CO_2_) and a Nikon PerfectFocus autofocus system. The set-up was operated by Micromanager 1.4.14. Images were acquired for 4 h at 2 min intervals. Cells were tracked manually and analyzed with the free Chemotaxis and Migration Tool from Ibidi (Munich, Germany) using ImageJ. Accumulated distance along the track and Euclidian distance were measured in μm, velocity was calculated as the accumulated distance divided by the time, and directionality was calculated as the ratio of Euclidian distance to accumulated distance.

### Transwell migration assay

1x10^5^ mature BMDCs (stimulated with 200 ng/ml LPS for 24 h) were seeded into a Transwell (Corning) with a pore size of 5 μm. BMDCs were allowed to migrate towards chemokine-free RPMI, or towards 250 ng/ml CCL19 or 250 ng/ml CCL21 in RPMI for 3 h at 37°C as described in [31]. Migrated BMDCs were harvested from the lower chamber of the Transwell and counted by flow cytometry for 60 s. 1x10^5^ cells in suspension were used as 100% control. Migration towards RPMI was defined as unspecific and was subtracted from the total migration towards chemokine to obtain the specific migration towards chemokine.

### 3D chemotaxis assay

Mature BMDCs were harvested and embedded into a 1.6 mg/ml collagen solution (PureCol, bovine collagen solution type I, Advanced Biomatrix), which was cast into a home-made chamber as described before [32]. After polymerization, the gel was covered with RPMI with or without 650 ng/ml CCL19 or CCL21 and sealed with a paraffin/wax mixture. Cells were allowed to migrate for 6 h at 37°C and 5% CO_2_. Images were taken every 4 min using the microscope system described for the 2D DC chemotaxis assay. At least 20 cells were manually tracked and analyzed per condition and experiment.

### FITC painting assay

200 μl of fluorescein isothiocyanate (FITC; 5 mg/ml in 1:1 dibutylphtalate/acetone) was applied onto the shaved abdomen of adult WT and Gadkin^-/-^ mice anesthetized by isoflurane. On the following day mice were sacrificed to isolate the draining inguinal lymph nodes. Lymph nodes (LNs) were suspended into single cells by first mashing them with the end of a syringe in a Petri dish and then applying the suspension to a 70 μm cell strainer. The suspension was centrifuged at 4°C for 5 min at 300 g. Cell pellets were resuspended in 200 μl FACS buffer and immediately measured on a FACSCantoII flow cytometer (Becton Dickinson) using the BD FACSDiva software. Data were quantified with FlowJo.

### DC homing assay

LPS-matured WT and Gadkin^-/-^ BMDCs were labeled with 10 μM TAMRA or 5 μM DDAO in suspension for 10 min at room temperature, washed once with medium and quenched for 30 min in medium. Prior to injection cells were washed twice with PBS. 20 μl of a 1:1 mixture of 10^6^ WT and Gadkin^-/-^ BMDCs labeled with different dyes were injected intracutaneously (i.c.) into a hind footpad of WT mice anaesthetized by isoflurane. Two days post-injection mice were sacrificed to isolate popliteal lymph nodes. Lymph nodes were suspended into single cells as described above, and cell composition was analyzed by flow cytometry as described above.

### Statistical analyses

Values are depicted as mean ± SEM (standard error of the mean). Statistical significance of data was analyzed by the indicated statistical tests using GraphPad Prism software, and are indicated in the following way: **** = p<0.0001; *** = p<0.001; ** = p<0.01; * = p<0.05. Data with arbitrary absolute values, like fluorescent intensities, which for technical reasons showed high variability between experiments, was normalized to the mean of all samples before performing statistics. The number of experimental replica or animals used is given as “N”.
